# Secretome of Senescent Adipose-Derived Mesenchymal Stem Cells Negatively Regulates Angiogenesis

**DOI:** 10.3390/ijms21051802

**Published:** 2020-03-05

**Authors:** Andrey Ratushnyy, Mariia Ezdakova, Ludmila Buravkova

**Affiliations:** Institute of Biomedical Problems, Russian Academy of Sciences, Khoroshevskoye Shosse 76a, 123007 Moscow, Russia; ratushkin@mail.ru (A.R.); devamarya@gmail.com (M.E.)

**Keywords:** adipose-derived mesenchymal stem cells (ASCs), replicative senescence, senescence-associated secretory phenotype (SASP), angiogenesis

## Abstract

Nowadays, paracrine regulation is considered as a major tool of mesenchymal stem cell (MSC) involvement in tissue repair and renewal in adults. Aging results in alteration of tissue homeostasis including neovascularization. In this study, we examined the influence of replicative senescence on the angiogenic potential of adipose-derived MSCs (ASCs). Angiogenic activity of conditioned medium (CM) from senescent and “young” ASCs was evaluated in chorioallantoic membrane (CAM) assay in ovo using Japanese quail embryos. Also, the formation of capillary-like tubes by human umbilical vein endothelial cells (HUVECs) in 3D basement membrane matrix “Matrigel” and HUVEC migration capacity were analyzed. Multiplex, dot-blot and gene expression analysis were performed to characterize transcription and production of about 100 angiogenesis-associated proteins. The results point to decreased angiogenic potential of senescent ASC secretome in ovo. A number of angiogenesis-associated proteins demonstrated elevation in CM after long-term cultivation. Meanwhile, VEGF (key positive regulator of angiogenesis) did not change transcription level and concentration in CM. Increasing both pro- (FGF-2, uPA, IL-6, IL-8 etc.) and antiangiogenic (IL-4, IP-10, PF4, Activin A, DPPIV etc.) factors was observed. Some proangiogenic genes were downregulated (*IGF1*, *MMP1*, *TGFB3*, *PDGFRB*, *PGF*). Senescence-associated secretory phenotype (SASP) modifications after long-term cultivation lead to attenuation of angiogenic potential of ASC.

## 1. Introduction

Mesenchymal stromal cells (MSCs) are a heterogeneous population of poorly differentiated stromal precursors with high proliferative activity and multilineage differentiation, which keeps them in demand for clinical use [1,2]. These days, the positive effects of MSCs are mainly attributed to their ability to produce a number of biologically active factors, including cytokines, exosomes, and extracellular matrix components [3,4,5,6]. The MSC secretome affects the microenvironment at damage area, promoting cytoprotection and tissue repair. These effects are of particular interest for the treatment of ischemia, where the stimulation of vascularization is crucial for the preservation of alive tissue and, therefore, for the prevention of fibrosis [7].

However, the properties of the cell population can vary significantly depending on the donor, tissue sources, and even individual cell clones. This fact complicates the comparison of the results and necessitates the study of each tissue-specific population separately [8]. Adipose tissue is one of the most perspective sources of MSCs since they can be obtained in sufficient amounts from patients using a minimally invasive procedure. Adipose-derived MSCs (ASCs) are considered a promising tool for various types of cell therapy and tissue engineering [9]. According to several authors, ASCs have some advantages over the bone marrow MSCs, including a greater number of precursors from the similar amount of the sample and an increased capability of proliferation, differentiation, and angiogenesis in vivo [10,11,12]. Application of ASCs resulted to increase in the number of vessels and blood flow restoration in damaged tissues after the limb ischemia [13,14,15] and myocardial infarction [16,17]. Neovascularization after administration of ASCs or conditioned medium (CM) was considered the main mechanisms of hepatic regeneration [18].

Due to their tissue niche role of maintaining homeostasis and auto-/paracrine regulation, MSCs are especially interesting from the point of view of cell senescence. With the activation of senescence, MSCs change their morphofunctional state. Irreversible arrest of the cell cycle occurs, the morphology, organelles activity, and gene expression are altered, γH2AX heterochromatin foci appear, and a number of other cell senescence markers are found. Senescent cells are able to maintain their viability and functional activity for a rather long period, continuing to interact with the microenvironment and providing local and systemic effects [19,20,21,22,23]. 

There are two main approaches for studying the effects of senescent cells. The first approach is a comparison of the cells obtained from young and elderly subjects. The study of primary cultures obtained from donors is of great practical importance for the clinics, since long-term cultures or other manipulations remain undesirable. Nevertheless, the obtained cultures are extremely heterogeneous, which makes it difficult to identify the effects of senescent cells, since their proportion in the tissue can vary greatly [24]. The second one is obtaining and investigation of senescent cells in vitro. For basic research, senescence induction in vitro is more informative, making it possible to obtain an almost homogeneous senescent cell culture and evaluate the physiological role thereof. 

In present paper we have compared the angiogenic potential of “young” and replicative senescent human ASCs.

## 2. Results

### 2.1. ASC Identification 

**Figure 1:**
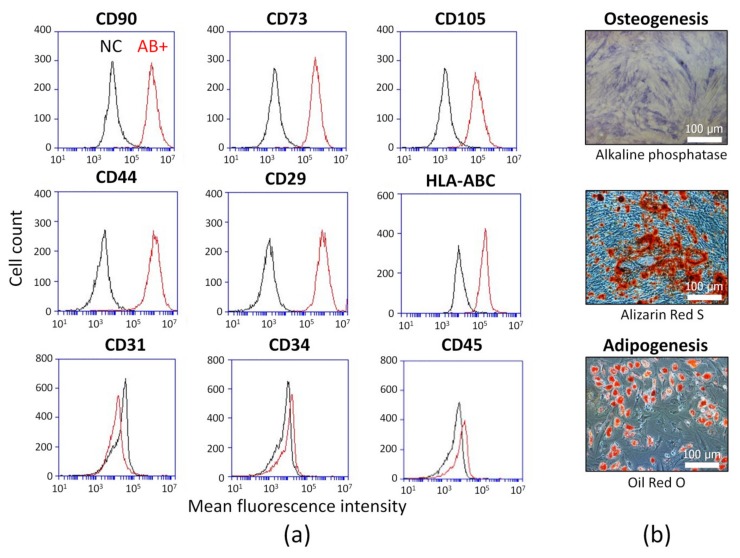
Biological characterization of adipose-derived mesenchymal stem cells (ASCs): (**a**) Representative flow cytometric plots of immunophenotype. AB+—ASCs were stained with antigen-specific fluorescent antibody. NC—negative control (ASCs were stained with matched fluorescent nonimmune antibody). Canonical ASC markers: CD90, CD105, CD73, CD44, CD29, HLA-ABC. Endothelial marker: CD31. Hematopoietic markers: CD34, CD45. (**b**) Differentiation capacity evaluation. ASCs were cultured in osteogenic differentiation medium and stained for alkaline phosphatase and alizarin red S. ASCs were cultured in adipogenic differentiation medium and stained with Oil red O.

The cells derived from human adipose tissue were analyzed. The fibroblast-like adhered cells with CD90+, CD73+, CD105+, CD44+, CD29+, HLA-ABC+, and CD31-, CD34-, CD45-immunophenotype were observed as demonstrated by flow cytometry (Figure 1a). Osteogenic and adipogenic potential were confirmed (Figure 1b). These data indicate that obtained cells are ASCs according to International Federation for Adipose Therapeutics and Science (IFATS) and International Society for Cell and Gene Therapy (ISCT) statements [25,26].

### 2.2. Cell Senescence Identification

Senescent ASCs were obtained by using long-term cultivation until the Hayflick limit and detection of cell senescence markers. 

**Figure 2:**
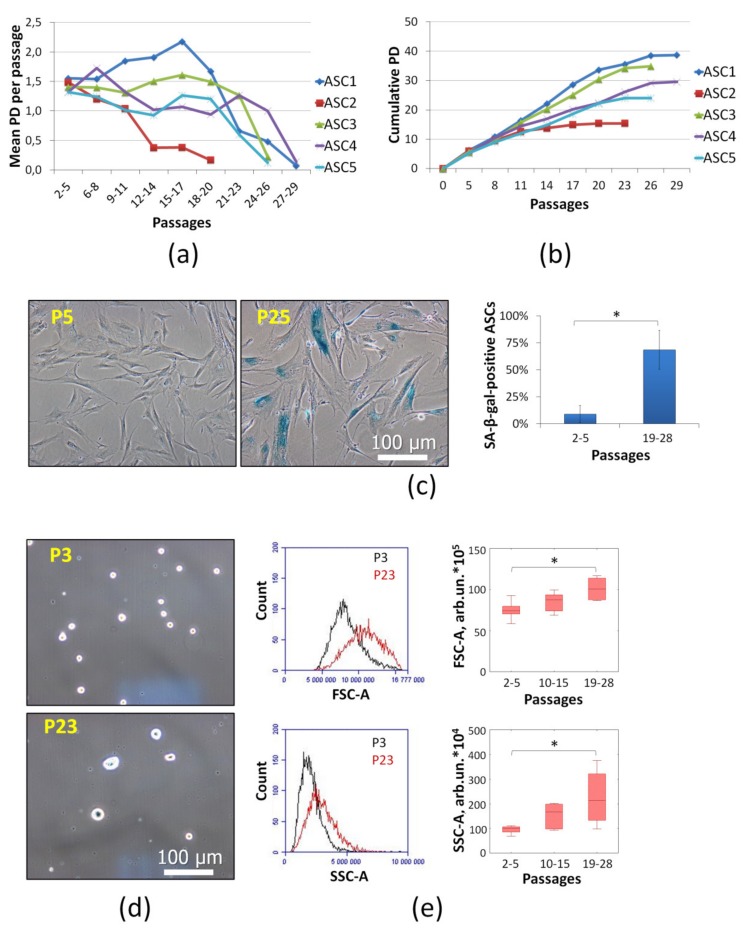
Senescence markers of long-term cultivated ASCs: (**a**) Proliferative activity was assessed as population doublings (PD) per passage (7 days). The values were averaged over several passages (*n* ≥ 3). ASC1-5 are cells isolated from different donors. (**b**) Cumulative PD was assessed as sum of PDs per passage. (**c**) Histochemical evaluation of senescence associated-β-galactosidase (SA-β-gal) activity in ASCs at early (P2-5) and late passages (P19-28). Representative images (light microscopy) and the share of SA-β-gal-positive ASCs. Data are shown as mean ± standard deviation (*n* ≥ 5, * *P* < 0.05). (**d**) Morphology of trypsinized ASCs (in suspension) at different passages (P3 and P23). Representative images (light microscopy). (**e**) Representative flow cytometry histograms of forward scattering (FSC-A) and side scattering (SSC-A) to illustrate size and cytoplasm vacuolization of ASCs, respectively; average ASC FSC-A (size) and SSC-A (cytoplasm vacuolization) at different passages. Data are shown as boxplots (median, ±Q1/Q3, ±min/max; *n* ≥ 8, * *P* < 0.05).

The main hallmark of cell senescence is irreversible cell cycle arrest, i.e., loss of proliferative properties. To evaluate ASC proliferation the mean population doubling (PD) per passage (7 days) of primary cell cultures from five donors were analyzed (Figure 2a). Long-term cultivation resulted in significantly decreased PD after 20 passages (140 days in culture). Cumulative PDs were about 16–40 (Figure 2b). Decreased proliferation point to increase senescent cells share in culture and spontaneous immortalization absence. ASC PDs were significantly differenced depending on the cell sample. For this reason, ASCs at late passages (p19-28) with decreased proliferative potential and other senescence markers were used.

In particular, senescent cells express senescence associated-β-galactosidase (SA-β-gal). The detection of SA-β-gal is a frequently used marker of cell senescence. This enzyme is a lysosomal hydrolase activated at pH 4. Nevertheless, it was shown SA-β-gal may be activated at pH 6 in senescent cells [27]. In this study, we displayed that long-term cultivated ASCs with decreased proliferation consist of SA-β-gal-positive cells (about 70% at late passages) (Figure 2c). This fact confirmed cell senescence. At early passages share of SA-β-gal-positive ASCs was very low.

Cell size and granularity increase are considered senescence signs as well [23,28,29]. These changes are associated with the active metabolism, including synthetic processes, without cell division and with accumulation of non-utilizable lipofuscin granules. Microphotographs of trypsinized (in suspension) ASCs clearly show that the cell size was increased at the late passages (P23) (Figure 2d). Microphotographs of adhesive ASCs at different passages (P5 and P25) demonstrated morphology alterations (Figure 2c). For ASC size and cytoplasm granularity evaluation flow cytometric forward scatter (FSC) and side scatter (SSC), respectively, were analyzed. The morphology of ASCs was altered at the late passages. Measured parameters were increased significantly (Figure 2e). All five cell cultures reached senescence, when the proliferation was compromised. The PD differences between ASC cultures did not affect the results of further assays significantly.

### 2.3. Angiogenic Potential of Senescent ASCs

**Figure 3:**
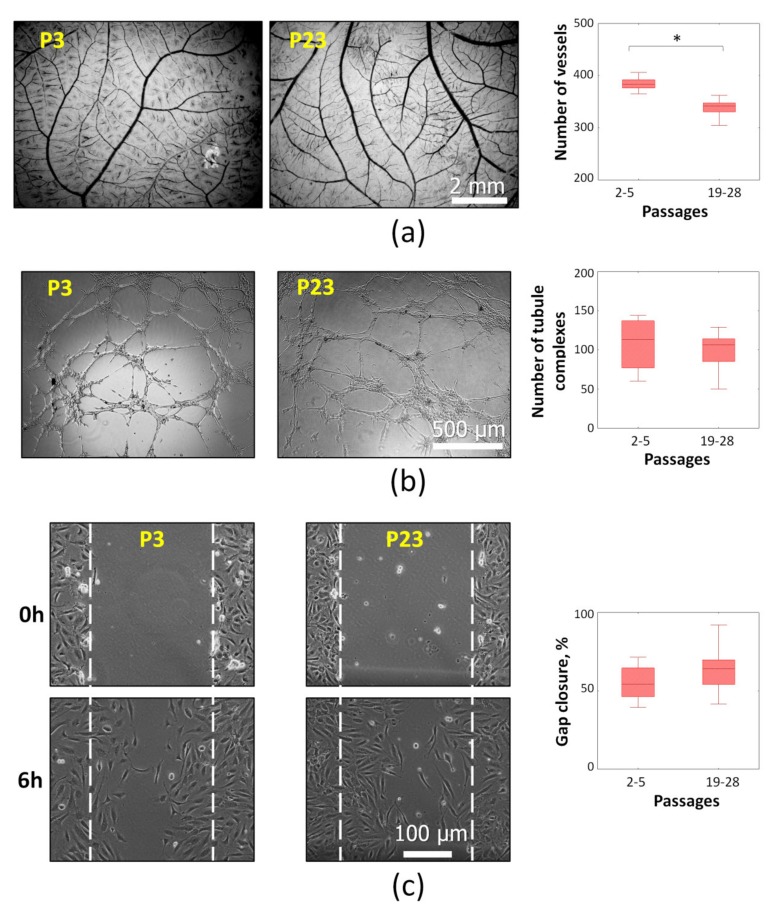
Angiogenic effects of ASC conditioned medium at different passages: (**a**) Angiogenesis in ovo. Representative images of chorioallantoic membrane (CAM) blood vessel network staining (light microscopy) and vessel number. (**b**) The capillary-like tube formation by HUVECs in vitro. Representative images of the capillary-like tubes in “Matrigel” (light microscopy) and number of tubule complexes. (**c**) Endothelial cells migration in “wound healing” assay. Representative images of “wound healing” (light microscopy) and gap closure (%) after 6 h. The gap closure was calculated as (1 − N/N0)*100%, where N—final wound area (6h after scraping) and N0—initial wound area (0 h after scraping). All data are shown as boxplots (median, ±Q1/Q3, ±min/max; *n* ≥ 4, * *P* < 0.05).

To assess angiogenic activity of CM from senescent (P19-28) and “young” (P2-5) ASCs, a set of experimental models was applied. The effect on the blood vessel growth in ovo was tested in chorioallantoic membrane (CAM) assay using Japanese quail embryo. This approach allows evaluating the CM angiogenic potential in complex model with many types of interacting cells which are necessary for the capillary network formation. After 24 h of exposure with CM from senescent ASCs a significant decreased vessel number was detected (Figure 3a).

Other models provided data about effect of CM to distinct angiogenesis-associated processes including capillary-like tube formation and endothelial cells (ECs) migration. The formation of capillary-like tubes by ECs (human umbilical vein endothelial cells—HUVECs) was evaluated by using 3D basement membrane matrix “Matrigel”. CM from “young” and senescent ASC did not provoke significant differences (Figure 3b). Nontargeted HUVEC migration was evaluated in cell monolayer using the in vitro “wound healing” assay. According to obtained results no differences were observed between CM from “young” and senescent ASCs (Figure 3c).

Thus, replicative senescence of ASCs results in reduced angiogenic potential of their secretome. This effect was observed on the blood vessel growth model in ovo, where various types of cellular and non-cellular elements mediating angiogenesis are present. However, no differences on the tubular structure formation in “Matrigel” and HUVECs migration were found.

### 2.4. Characterization of Angiogenesis-Associated Secretome of Senescent ASCs

**Figure 4:**
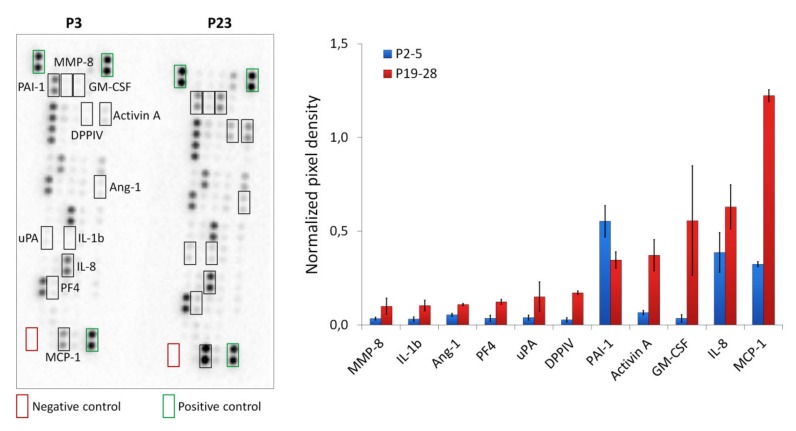
Evaluation of human angiogenesis-associated proteins in conditioned medium of “young” (P2-5) and senescent (P19-28) ASCs using Proteome Profiler Human Angiogenesis Array Kit (R&D). Representative image of the dot blot and protein production by different groups of ASCs are shown. Densitometric analysis of certain analytes in antibody arrays was quantified using ImageLab software (Bio-Rad). The proteins that significantly (*P* < 0.05) changed more than 1.5 times are demonstrated. The data were normalized to positive control and are shown as mean ± standard deviation (*n* = 4).

To characterize the profile of ASC CM, we analyzed 55 human angiogenesis-related proteins using Proteome Profiler Human Angiogenesis Array Kits. The results indicated that concentration of 11 proteins in senescent ASC CM differed from “young” cell CM significantly more than 1.5 times (Figure 4). MMP-8, IL-1b, Ang-1, PF4, uPA, DPPIV, Activin A, GM-CSF, IL-8, MCP-1 were increased and PAI-1 was decreased.

**Figure 5:**
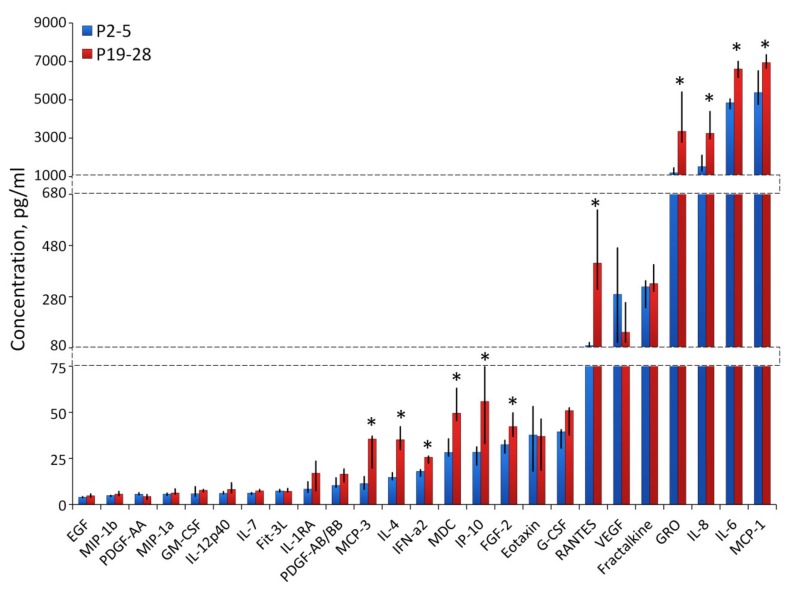
Evaluation of human cytokine concentration in conditioned medium of “young” (P2-5) and senescent (P19-28) ASCs by multiplexed fluorescent bead-based immunoassay detection. Data are shown as median ± min/max; *n* ≥4, * *P* < 0.05.

Forty-one cytokines in CM were measured by multiplexed fluorescent bead-based immunoassay detection (Figure 5). Among the 41 analyzed cytokines, the pro-inflammatory interleukins—IL-6, IL-8 and chemokines—MCP-1, GRO (CXCL1) were most represented. The concentration of these cytokines was increased significantly at the late passages (P19-28). Also, we observed enhanced level of RANTES (CCL5), FGF2, IP10, MDC, IFNa2, IL-4, MCP-3.

**Figure 6:**
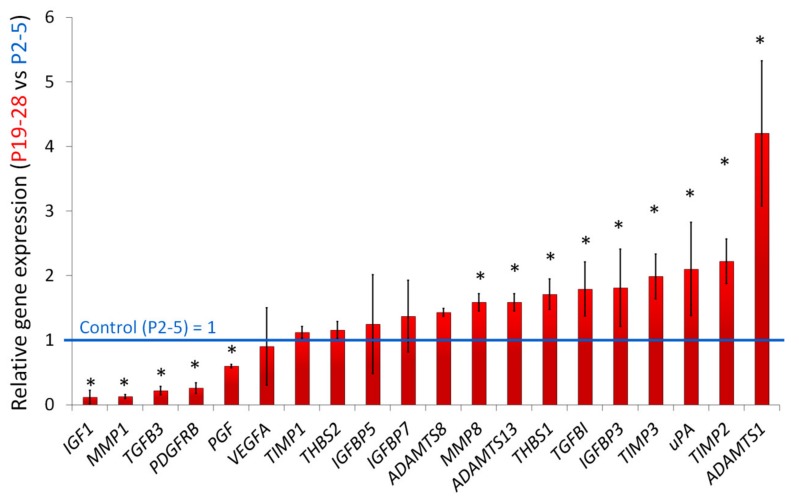
Differential angiogenesis-associated gene expression in senescent ASCs (senescent (P19-28) cells vs “young” (P2-5) cells). The data are shown as mean ± standard deviation of relative expression, *n* ≥ 3, * *P* < 0.05 (differences more than 1.5 times). Gene expression was evaluated by the 2^−ΔΔCt^ method.

Gene expression of secreted proteins associated with angiogenesis was analyzed (Figure 6). Significant differences (more than 1.5 times, *p* ≤ 0.05) between senescent and young ASCs were shown. *IGF1*, *MMP1*, *TGFB3*, *PDGFRB*, *PGF* were downregulated and *MMP8*, *ADAMTS13*, *THBS1*, *TGFBI*, *IGFBP3*, *TIMP3*, *uPA*, *TIMP2*, *ADAMTS1* were upregulated. It is noted the products of the *IGF1*, *MMP1*, *TGFB3*, *PDGFRB*, *PGF* genes exhibit proangiogenic activity. The mRNA level of *IGF1*, *MMP1*, *TGFB3*, *PDGFRB* in senescent ASCs were 5–10 times less in comparison to “young” ASCs. No significant change in *VEGFA* expression was detected. Upregulated genes including both proangiogenic (*MMP8*, *ADAMTS13*, *IGFBP3*, *uPA*) and antiangiogenic (*THBS1*, *TGFBI*, *TIMP3*, *TIMP2*, *ADAMTS1*) secreted factors were identified.

Thus, replicative senescence leads to an increased paracrine activity of human ASCs. In particular, a significant increased production of inflammatory cytokines (IL-6, IL-8, MCP-1, RANTES, etc.) was demonstrated. Many cytokines with proangiogenic activity were enhanced in senescent ASCs. On the other hand, a number of secreted proteins with antiangiogenic activity (such as IL-4, IFN-a2, IP10, IL-1b, PF4, DPPIV, Activin A, etc.) were increased too. Downregulation of some growth factor and protease genes (*IGF1*, *MMP1*, *TGFB3*, *PDGFRB*, *PGF*) associated with positive regulation of angiogenesis was noted. There were no significant differences of VEGF concentration and expression at early (P2-5) and late passages (P19-28). 

## 3. Discussion

With aging, the regenerative capabilities of the tissues that are largely due to the activity of adult stem cells are decreased. In the elderly, the risks associated with ischemic damages grow up, and the recovery is more difficult. This may be associated with a violation in angiogenic processes. On the one hand, impaired neovascularization suppresses regeneration of injured tissues; on the other hand, decreased angiogenesis may inhibit tumor growth. It is known that cancer is aging associated pathology and the risk of most primary neoplasia is increased in a geriatric population. However, growth of tumors in elderly tends to be less rapid. Some authors suggest that decreased neovascularization in a geriatric population may restrict the growth of some tumors. Nevertheless, the studies reflect complex and multifactorial processes and require further analysis [30,31]. 

Our results indicate a decreased angiogenic potential of senescent ASCs in a long-term culture. Similar data were demonstrated in a few studies of ASCs isolated from the tissues of young and elderly donors [32,33]. It was revealed that the donor age impaired angiogenic capacities of human ASCs in a mouse model of ischemic hindlimb [33]. Another study demonstrated a significant decrease in the vascular count when the cells from elderly donors were used in an in vivo “Matrigel” subcutaneous implant in the aged mice (18 months). At the same time, an evaluation of the effect of CM on the total length of capillary-like structures in “Matrigel” in vitro demonstrated mild differences of no more than 10% [32]. Nakamura et al. concluded that the donor age had no effects on the angiogenic properties of ASC CM, although they noted the presence of senescent cells in cultures obtained from elderly donors. It is worth noting that the authors used simplified models close to the in vivo conditions. ASC-CM-induced HUVEC tube formation was evaluated capacity in vitro only [34]. Thus, based on our results and the data of experiments using ASCs from the donors of various ages, we can conclude that senescent ASCs have a lesser angiogenic activity versus "young" cells. Moreover, this effect is fully manifested only in the complex models close to in vivo conditions, where many cellular and non-cellular elements involved in angiogenesis are present simultaneously. With elementary models, such as “Matrigel” and others, the effect is weak or absent. 

Angiogenesis is a complex physiological process that provides the growth and development of the circulatory system due to the proliferation and migration of endothelial cells that requires a sequential activation of stimulating and inhibitory signals [35,36]. In the angiogenesis research in vitro, the effect on the main stages of new vascular formation, including EC proliferation and migration, as well as their capability to form three-dimensional structures in a monoculture, is usually studied. Our results demonstrated the absence of significant changes in HUVEC undirected migration and tubular complexes formation after application of CM from senescent and "young" ASCs. The data are consistent with the results on the vascular endothelial growth factor (VEGF) concentration which plays a leading role in the induction of proliferation and migration of endotheliocytes, as the primary link of angiogenesis [37]. Previous studies have shown a linear relationship between the VEGF and FGF-2 concentrations and the formation of tubular complexes by endothelial cells [38]. However, the interaction of endothelial cells with pericytes, smooth muscle cells, fibroblasts, and the extracellular matrix is also crucial for the angiogenesis processes [39,40]. In this regard, to get a comprehensive picture of changes in the senescent MSC angiogenic potential, it is necessary to perform analysis using those techniques that are able to evaluate the effects not on the separate stages, but on the vascular formation as a whole, including all stages of angiogenesis. 

Senescence-associated secretory phenotype (SASP) is the most important case of disturbance of cell communication, which leads to various consequences in the surrounding tissues [41]. The paracrine profile comprises hundreds of secreted factors, including proinflammatory cytokines, chemokines, growth factors, and proteases [41,42,43]. The exact composition may vary depending on the cell type and the senescence induction method. So, it be found most, but not all, human and mouse fibroblast strains secrete biologically active VEGF at senescence [42]. According to our data, replicative senescence results mainly in an increased paracrine activity of ASCs. Upregulation of both pro- (FGF-2, uPA, IL-6, IL-8 etc.) and antiangiogenic (IL-4, IP-10, PF4, Activin A, DPPIV, etc.) factors was observed. Remarkably, most of the mediators that influence angiogenesis implement their function through VEGF [44,45,46,47]. Probably, the effects of activators are compensated by the effects of inhibitors. Thus, IL-4, IFN-a, PF4 and DPPIV counteract the angiogenic effect of VEGF and FGF, as was previously demonstrated using in vitro methods. Decreased migration activity of endothelial cells and the formation of capillary-like tubes in “Matrigel” have been shown [44,45,46,47,48,49,50]. 

In vivo, periendothelial cells secrete various vasoactive peptides, growth factors, and cytokines that regulate the proliferation, survival, and migration of endothelial cells, vascular tube branching, and vascular permeability [51]. Violation of angiogenic processes during aging can be associated both with a decreased expression of pro-angiogenic factors and the induction of angiogenesis inhibitors expression [52], endothelial progenitor cells dysfunction [53], and other factors. It is possible that the differences between in vivo/in ovo and in vitro models are denoted by the direct effects of ASC angiogenesis inhibitors on periendothelial cells.

Having analyzed the data, we found a decreased expression of genes whose products are involved in vascular formation (*PGF*, *PDGFRB*, and *TGFB3*), and a significantly increased expression of angiogenesis inhibitor genes (*TIMP3*, *TIMP2*, and *ADAMST1*). Matrix metalloproteases (MMPs) play important role in multiple physiological and pathological processes due to the processing of matrix proteins. There is a biological mechanism for limitation of tissue proteolysis caused by active MMPs by tissue inhibitors of metalloproteases (TIMPs). Dong-Wan So et al. demonstrated that TIMP-2 has the ability to directly inhibit endothelial cell proliferation in vitro and vascular growth in vivo [54]. TIMP-3 is a powerful anti-angiogenic protein that blocks VEGF binding to VEGF receptor-2 and inhibits endothelial cell proliferation, migration, and capillary-like tube formation. [55]. ADAMTS1 can suppress angiogenesis, as demonstrated by various in vitro and in vivo models. Nathan V. Lee et al. demonstrated that ADAMTS1 significantly blocks the phosphorylation of VEGFR2 via direct binding and sequestration of VEGF165, which subsequently leads to a decreased proliferative activity of endothelial cells [56]. No changes in the amount of product in the CM may indicate the presence of post-translational mechanisms to reduce the secretion of pro- and antiangiogenic MSC factors during aging. The level of VEGF mRNA changes slightly with age, which also correlates with its concentration in the CM.

Thus, the replicative senescence of ASCs results in a significant modification of the profiles of secreted factors involved in angiogenesis. A simultaneous increase in the production of most pro- and antiangiogenic factors and a change in gene expression occur simultaneously. However, VEGF production remains unchanged. Nevertheless, a secretome modification results in a decreased angiogenic potential of ASCs.

## 4. Materials and Methods 

### 4.1. ASCs Isolation and Expansion

Adipose tissue samples were obtained under the Scientific Agreement from multidisciplinary clinic (Moscow, Russia) after elective liposuction procedures performed with local anesthesia from five female donors (age range 35–45 years old) with written informed consent. Adipose tissue was processed using the guidelines specifically approved by the Biomedicine Ethics Committee of the Institute of Biomedical Problems, Russian Academy of Sciences (Physiology Section of the Russian Bioethics Committee, Russian Federation National Commission for UNESCO, Permit #314/МCK/09/03/13). Adipose stromal cells (ASCs) were isolated using a standard method described by Zuk et al. [57] as modified by Buravkova et al. [58]. 

The isolated cells were stained with antibody against stromal markers CD90, CD73, CD105, CD44, CD29, HlA-ABC, CD31, CD34, CD45 and were analyzed using an Accuri C6 flow cytometer (BD Biosciences, San Jose, CA, USA). To induce osteogenic differentiation, complete α-MEM was supplemented with 10−8 М dexamethasone, 10 mМ glycerol-2-phosphate, and 0.2 mМ L-ascorbic acid 2-phosphate (Sigma, St. Louis, MO, USA). Osteogenic differentiation was confirmed with Alkaline phosphatase kit (Sigma-Aldrich, St. Louis, MO, USA) and Alizarin red staining of the mineralized matrix components (Millipore, Bedford; MA, USA). To induce adipogenic differentiation, the medium was supplemented with 0.5 mМ isobutyl methylxanthine, 1 μM dexamethasone, 10 μg/mL insulin, and 200 μM indomethacin (Sigma, St. Louis, MO, USA). Adipogenic differentiation was assessed by the evaluation of cytoplasmic Oil-Red-O-stained lipid droplets (Millipore, Bedford, MA, USA).

The cells were expanded in α-MEM (Gibco, Life Technologies, Carlsbad, CA, USA) with 50 U/mL penicillin-streptomycin (PanEco, Moscow, Russia), and 10% fetal bovine serum (FBS) (HyClone, Logan, UT, USA) at standard conditions (5% CO_2_, 37 °C). Subculture was done at 80–90% confluence of the cell layer. Long-term cultivation was continued until replicative senescence was observed in the culture. 

### 4.2. Cell Senescence Identification

Proliferative activity was assessed as population doublings (PDs) per passage (7 days). PD was estimated as follows: PD = [log (N/N0)]/log 2, where N0 and N are initial and final cell quantities [59]. The values were averaged over several passages [60,61,62]. Cumulative PD was assessed as sum of PDs per passage. Cells were seeded at standard density (3 × 10^3^ cells/cm^2^). Cell number was determined using a hemocytometer at day 0 and day 7.

Expression of senescence associated-β-galactosidase (SA-β-gal) activity at pH 6.0 was estimated with the Senescence Cells Histochemical Staining Kit (Sigma, St. Louis, MO, USA) following the manufacturer’s protocol. We analyzed five view fields in each experimental point using an Eclipse TiU phase-contrast microscope (Nikon, Japan). Cell count was performed with Sigma ScanPro 5.0 Image Analysis Software (SPSS Inc., Chicago, IL, USA). Positive and negative cells were counted and the percentage of SA-β-gal^+^ cells was calculated.

For cell size and structure analysis flow cytometric forward scatter (FSC) and side scatter (SSC) density plots were applied (Accuri C6 flow cytometer, BD Biosciences, San Jose, CA, USA). The enlargement of cells and increase of the granularity are considered as specific features of cell senescence as well [28,29].

### 4.3. Isolation and Culture of ECs

Cryopreserved human umbilical vein endothelial cell (HUVEC) samples were provided by the Cryocenter Cord Blood Bank (Moscow, Russia), as part of a Scientific Agreement. The cells were cultured in 199 medium (M199) (Gibco, Life Technologies, Carlsbad, CA, USA) supplemented with 10% FBS (HyClone, Logan, UT, USA), 200 mg/mL EC growth factor (Sigma-Aldrich, St. Louis, MO, USA), 2 mM glutamine (Gibco, Life Technologies, Carlsbad, CA; USA), 1 mM sodium pyruvate (Gibco, Life Technologies), 50 U/mL penicillin, and 50 mg/mL streptomycin under 5% CO_2_, 37 °C in a CO_2_-incubator (Sanyo, Osaka, Japan). HUVECs formed a typical confluent monolayer of elongated and polygonal cells positively stained with antibody against CD31–a marker of ECs.

### 4.4. Chorioallantoic Membrane Assay in Ovo

ASC angiogenic activity was evaluated in chorioallantoic membrane (CAM) assay using Japanese quail embryos [63]. The fertilized eggs were placed into the incubator and kept at 37 °C for 6 days. Then, a window was made in the shell and 30 mL of ASC conditioned medium were added. The windows were sealed with medicinal glue BF-6 (Vertex, Saint-Petersburg, Russia) and the incubation was continued for extra 24 h. The embryos were fixed with 4% paraformaldehyde/2% glutaraldehyde solution in PBS and stained with Carracci’s hematoxylin. Then, the vascularization of CAM was analyzed by morphometric analysis using AngioQuant software (www.cs.tut.fi). 

### 4.5. Capillary-Like Tube Formation

ASC angiogenic activity in vitro was examined using model of capillary-like tube formation in “Matrigel” according to the manufacturer’s protocol. HUVECs from second to fourth passages were seeded in 96-well plates coated with growth factor reduced “Matrigel” (Corning, Corning, NY, USA) in concentration 2 × 10^4^ cells per well. Conditioned medium was diluted with full M199 1:1. At least two wells were used for each sample of ASC conditioned medium. M199 serum-free was utilized as negative control; M199 with 10% FBS and VEGF (25 ng/mL) served as positive control. Plates were placed into CO_2_-incubator at 37 °C and capillary-like structures were examined after 15 h under the phase contrast microscope (Leica, Wetzlar, Germany). Total number of tubular complexes was counted with Image Processing Software—Image-Pro Plus (Media Cybernetics, Inc., Rockville, MD, USA). 

### 4.6. Nontargeted Cell Migration Assay (“Wound Healing”)

Nontargeted EC migration was evaluated in cell monolayer at a cell density of 10^4^ cells/cm^2^ using the in vitro “scratch” assay [64]. ECs were scratched with a sterile pipette tip to create a “wound” approximately 0.8–1.0 mm wide. Culture medium was replaced with full M199 diluted ASC conditioned medium with 1:1. To estimate the wound closure, serial digital images were captured with a Nikon Eclipse Ti-U microscope (Nikon, Tokyo, Japan) at specific time intervals (0 and 6 h) after the scratch. The images were analyzed using NIS-Elements software (Nikon, Tokyo, Japan) which measured the width of the scratch at previously marked points (five per Petri dish) along its length. The gap closure was calculated as (1 − N/N0)*100%, where N—final wound area and N0—initial wound area.

### 4.7. Analysis of Proteins Secreted by ASCs 

The conditioned medium (CM) was collected before reseeding, centrifuged at 2500 g to remove cell debris, and stored at −80 °C (low temperature freezer, Sanyo, Osaka, Japan).

To detect 55 human angiogenesis-related proteins (Activin A, ADAMTS-1, Angiogenin, Angiopoietin-1, Angiopoietin-2, Angiostatin, Amphiregulin, Artemin, Coagulation Factor III, CXCL16, DPPIV, EGF, EG-VEGF, Endoglin, Endostatin/Coll18, Endothelin-1, FGF-1, FGF-2, FGF-4, FGF-7, GDNF, GM-CSF, HB-EGF, HGF, IGFBP-1, IGFBP-2, IGFBP-3, IL-1b, IL-8, LAP (TGF-b1), Leptin, MCP-1, MIP-1a, MMP-8, MMP-9, NRG1-b1, PTX3, PD-ECGF, PDGF-AA, PDGF-AB/PDGF-BB, Persephin, PF-4, PIGF, Prolactin, Serpin B5, Serpin E1, Serpin F1, TIMP-1, TIMP-4, TSP-1, TSP-2, uPA, Vasohibin, VEGF, VEGF-C), CM was analyzed using Proteome Profiler Human Angiogenesis Array Kit (R&D Systems, Inc., Minneapolis, MN, USA) according to the manufacturer’s instructions. The data were analyzed using Image Lab Software Version 5.0 (Bio-Rad, Hercules, CA, USA).

Forty-one cytokines were measured by multiplexed fluorescent bead-based immunoassay detection (MILLIPLEX^®^ MAP system, Merck Millipore, Darmstadt, Germany) according to the manufacturer’s instructions, using a Human Cytokine/Chemokine panel (41-plex). The panel contained antibody-conjugated beads for following cytokines and chemokines: EGF, FGF-2, Eotaxin, TGF-a, G-CSF, Fit-3L, GM-CSF, Fractalkine, IFN-a2, IFNg, GRO, IL-10, MCP-3, IL-12p40, MDC, IL-12p70, PDGF-AA, IL-13, PDGF-AB/BB, IL-15, sCD40L, IL-17A, IL-1RA, IL-1a, IL-9, IL-1b, IL-2, IL-3, IL-4, IL-5, IL-6, IL-7, IL-8, IP-10, MCP-1, MIP-1a, MIP-1b, RANTES, TNFa, TNFb, VEGF. For each assay, the curve was derived from various concentrations of the cytokine standards assayed in the same manner as conditioned medium samples. All samples were measured undiluted.

### 4.8. Quantitative PCR Analysis

To evaluate gene expression, total RNA was extracted with QIAzol Reagent (Qiagen, Hilden, Germany) and purified by the phenol/chloroform technique. The quality and concentration of RNA samples were estimated by using a Nanodrop ND-2000c (Thermo Scientific, Waltham, MA, USA). Reverse transcription was performed using the QuantiTect Reverse Transcription Kit (Qiagen, Hilden, Germany) according to the manufacturer’s protocol. Expression of the genes *IGF1*, *MMP1*, *TGFB3*, *PDGFRB*, *PGF*, *VEGFA*, *TIMP1*, *THBS2*, *IGFBP5*, *IGFBP7*, *ADAMTS8*, *MMP8*, *ADAMTS13*, *THBS1*, *TGFBI*, *IGFBP3*, *TIMP3*, *uPA*, *TIMP2*, *ADAMTS1* was analyzed using Qiagen primers (Qiagen, Hilden, Germany). The expression levels of five housekeeping genes (*ACTB*, *B2M*, *GAPDH*, *HPRT*, and *RPLP0*) were used for reference. Polymerase chain reaction was performed using the Mx300P system (Stratagene, San Diego, CA, USA). Normalized gene expression was calculated with the 2^−ΔΔCt^ method [65].

### 4.9. Statistical Analysis

A minimum of three independent experiments were performed for each assay. Analysis of group differences was performed by nonparametric Mann–Whitney test for independent samples using SPSS 14.0 software (SPSS Inc., Chicago, IL, USA). A level of *P* < 0.05 was accepted as statistically significant.

## Abbreviations

MSCs	Mesenchymal stem cells
ASCs	Adipose-derived mesenchymal stem cells
IFATS	International Federation for Adipose Therapeutics and Science
ISCT	International Society for Cell and Gene Therapy
CD	Cluster of differentiation
PD	Population doubling
FSC	Forward scatter
SSC	Side scatter
SA-β-gal	Senescence associated-β-galactosidase
CM	Conditioned medium
CAM	Chorioallantoic membrane
HUVECs	Human umbilical vein endothelial cells
ECs	Endothelial cells
SASP	Senescence-associated secretory phenotype
FBS	Fetal bovine serum
